# The role of the precuneus in metaphor comprehension: evidence from an fMRI study in people with schizophrenia and healthy participants

**DOI:** 10.3389/fnhum.2014.00818

**Published:** 2014-10-16

**Authors:** Nira Mashal, Tali Vishne, Nathaniel Laor

**Affiliations:** ^1^School of Education, Bar-Ilan UniversityRamat-Gan, Israel; ^2^Multidisciplinary Brain Research Center, Bar-Ilan UniversityRamat-Gan, Israel; ^3^Tel Aviv-Brull Community Mental Health CenterTel Aviv, Israel; ^4^Child Study Center, Yale UniversityNew Haven, CT, USA

**Keywords:** schizophrenia, novel metaphors, precuneus, language, fMRI

## Abstract

Comprehension of conventional and novel metaphors involves traditional language-related cortical regions as well as non-language related regions. While semantic processing is crucial for understanding metaphors, it is not sufficient. Recently the precuneus has been identified as a region that mediates complex and highly integrated tasks, including retrieval of episodic memory and mental imagery. Although the understanding of non-literal language is relatively easy for healthy individuals, people with schizophrenia exhibit deficits in this domain. The present study aims to examine whether people with schizophrenia differentially recruit the precuneus, extending to the superior parietal (SP) cortex (SPL), to support their deficit in metaphor comprehension. We also examine interregional associations between the precuneus/SPL and language-related brain regions. Twelve people with schizophrenia and twelve healthy controls were scanned while silently reading literal word pairs, conventional metaphors, and novel metaphors. People with schizophrenia showed reduced comprehension of both conventional and novel metaphors. Analysis of functional connectivity found that the correlations between activation in the left precuneus/SPL and activation in the left posterior superior temporal sulcus (PSTS) were significant for both literal word pairs and novel metaphors, and significant correlations were found between activation in the right precuneus/SPL and activation in the right PSTS for the three types of semantic relations. These results were found in the schizophrenia group alone. Furthermore, relative to controls, people with schizophrenia demonstrated increased activation in the right precuneus/SPL. Our results may suggest that individuals with schizophrenia use mental imagery to support comprehension of both literal and metaphoric language. In particular, our findings indicate over-integration of language and non-language brain regions during more effortful processes of novel metaphor comprehension.

## Introduction

Patients with schizophrenia demonstrate pervasive deficits in processing different pragmatic aspects of language, and in particular they show impairments in understanding proverbs, irony, and metaphors (Rapp, 2009). Comprehension of figurative language relies on effortful cognitive processes in which the non-literal message of the utterance is extracted. People with schizophrenia tend to interpret proverbs literally, a phenomenon termed “concretism”, and clinicians regard proverb interpretation as a potential tool in the diagnosis of schizophrenia (Reich, 1981). Some researchers have suggested that schizophrenia is associated with more general difficulties in abstract thinking (for a review see Thoma and Daum, 2006). The present study focuses on the challenges that people with schizophrenia experience when processing metaphor comprehension, especially novel metaphors.

Metaphors do not constitute a homogenous class of expressions but instead there is a continuum from idioms (dead metaphors) at one end to novel metaphors (live metaphors) at the other end (Fraser, 1998), with metaphors of different levels of conventionality in between. That is, some conventional metaphors had once been novel but due to repeated use have lost their metaphoricity. Metaphor comprehension is said to depend on level of conventionality (Glucksberg and Keysar, 1990; Giora, 1997; Giora and Fein, 1999; Bowdle and Gentner, 2005). The Career of Metaphor model (Bowdle and Gentner, 2005) argues that a newly created metaphor is comprehended via a comparison process, whereas a conventional metaphor is understood via a categorization process. Because the meanings of conventional metaphors are already stored in long term memory (i.e., they have been lexicalized), they are retrieved directly from the mental lexicon or via a previously created abstract metaphoric category. Unlike the comprehension of conventional metaphors, comprehension of novel metaphors involves an on-line effortful process of extraction and comparison of features.

Computing the metaphorical interpretation of an utterance relies on additional cognitive processes. Appraisal of the meaning of figurative language seems to be associated with the development of the ability to evoke mental images. Accordingly, school-aged children provide less sophisticated, less comprehensive, and more concrete mental images of idiom content than do adults (Nippold and Duthie, 2003). Behavioral evidence concerning the role of mental imagery in the comprehension and memory of idioms suggests that when people interpret idioms they construct a general mental image that is strongly constrained by conceptual mappings between base and target domains (for a review see Gibbs and O’Brien, 1990). For example, the mental image associated with *spill the beans* is derived from the conceptual mapping between the image of a mind as a container and the image of ideas as physical entities (Lakoff, 1987). This mapping evokes the image of taking ideas out of the physical container of the mind. According to Gibbs and O’Brien (1990), these images are unconscious, automatic, and independent of modality. With respect to this notion, Bottini et al. (1994) noted that the retrieval of information from episodic memory as well as mental imagery may be necessary to overcome the denotative violation inherited in metaphoric language.

It has been suggested that deficient language comprehension in schizophrenia is associated with right hemisphere involvement (e.g., Kircher et al., 2002; Mitchell and Crow, 2005; Bleich-Cohen et al., 2009; for a review see Rapp, 2009). According to Mitchell and Crow (2005), the abnormalities in language processing that are typical of schizophrenia reflect activation in right hemisphere homolog regions of key left hemisphere language regions. Furthermore, Mitchell and Crow (2005) argued that these functional changes indicate the loss or reversal of lateralized activation of brain regions associated with particular components of language processing. Although there is behavioral evidence of impairments in non-literal language comprehension in schizophrenia (de Bonis et al., 1997; Drury et al., 1998; for a review see Rapp, 2009; but see also Titone et al., 2002), only few neuroimaging studies tested metaphoric processing in this population (Kircher et al., 2007; Mashal et al., 2013).

If the right hemisphere is deficient in schizophrenia (Mitchell and Crow, 2005), and since there is some evidence suggesting that processing of novel metaphors involves the right hemisphere (Mashal et al., 2005, 2007; Schmidt et al., 2007; Pobric et al., 2008; Mashal and Faust, 2009, but see Rapp et al., 2004, 2007), it is especially intriguing to test novel metaphor processing in schizophrenia. Kircher et al. (2007) found disrupted brain activation during an implicit task of metaphor processing in people with schizophrenia. Participants silently read novel metaphoric sentences (e.g., *the lovers’ words are harp sounds*) as well as matching literal sentences (e.g., *the lovers’ words are lies*), and then decided whether the sentence had a positive or a negative connotation. People with schizophrenia demonstrated increased activation in the left inferior frontal gyrus (IFG) while processing novel metaphors, whereas healthy participants demonstrated stronger signal changes in the right superior/middle temporal gyrus. Interestingly, the severity of concretism, as rated with the Positive and Negative Syndrome Scale (PANSS), was negatively correlated with left IFG activation, suggesting that activation of this region contributes to concrete thinking in schizophrenia. In a recent fMRI study (Mashal et al., 2013), people with schizophrenia who were asked to silently read novel metaphors demonstrated increased activation in left middle frontal gyrus (MFG) relative to processing of meaningless word pairs. This pattern of activation differed from enhanced brain activation in the right IFG observed in healthy controls. Thus, reversed lateralization patterns were documented in schizophrenia. These results suggest that inefficient processing of novel metaphors in schizophrenia may involve compensatory recruitment of additional brain regions, such as the left MFG, a region known to be involved in executive functioning, and specifically in working memory (e.g., Braver et al., 1997; Jha and McCarthy, 2006). Furthermore, direct comparison between the people with schizophrenia and healthy adults on processing of literal expressions and novel metaphors relative to a baseline condition revealed greater activation in left precuneus in the schizophrenia group.

The precuneus has been studied extensively over the past decade as a central hub of the default mode network (DMN), which typically shows deactivation compared to rest for sensory motor tasks in healthy participants (e.g., Fransson, 2005; Cavanna and Trimble, 2006; Fransson and Marrelec, 2008; Margulies et al., 2009; Zhang and Li, 2012). It has been observed that tasks that demand much attention are associated with decreased activity in the DMN (e.g., Mazoyer et al., 2001). The precuneus is interconnected with both cortical and subcortical regions. It is specifically connected to parietal areas, including the inferior and superior parietal (SP) cortex and the intraparietal sulcus, which have been associated with processing of visuo-spatial information (Selemon and Goldman-Rakic, 1988). Tracer injection studies in non-human primates have shown that the extra-precuneus cortico-cortical connections include the supplementary motor cortex, dorsal premotor area, anterior cingulate, and language related areas such as the prefrontal cortex (BA 8, 9, and 46), as well as the posterior superior temporal sulcus (PSTS) (for a review see Cavanna and Trimble, 2006). These widespread connections with frontal and temporal regions suggest that the precuneus may be involved in a variety of highly integrated and associative behavioral functions. The precuneus has been linked with language related tasks at word (e.g., Kouider et al., 2010) and sentence level comprehension (Whitney et al., 2009). Reviewing 100 studies, Price (2010) concluded that the comparison of comprehensible and incomprehensible sentences is associated with activation in four core regions including the precuneus, the anterior and posterior parts of the left middle temporal gyrus (MTG), bilateral anterior temporal poles, and the left angular gyrus. These regions were also identified in a meta-analysis of 120 studies (Binder et al., 2009) that pointed out seven brain regions engaged in semantic processing, including the posterior cingulate extending to the precuneus.

The precuneus has also been linked to episodic memory retrieval (Shallice et al., 1994), processing of mental imagery (Hassabis et al., 2007; Johnson et al., 2007; Burgess, 2008), and visuo-spatial memory functions (Vincent et al., 2006; Epstein et al., 2007). Previous studies have found that the retrieval of contextual associations is related to activation in the posterior precuneus and left prefrontal cortex. Lundstrom et al. (2005) suggested that the posterior precuneus is activated during regeneration of previous contextual associations and that the left lateral inferior frontal cortex is engaged in explicit retrieval as well as in integration of the contextual associations. Thus, the precuneus (together with inferior frontal cortex) is implicated in the recollection of past experiences. According to Binder et al. (2009), the precuneus is involved primarily in encoding episodic memories but at the same time it is consistently activated in semantic tasks, as it stores meaningful experiences together with their related associations in order to guide future behavior.

Evidence regarding the role of the precuneus in metaphor comprehension is mixed. Data from an fMRI study with healthy participants showed prominent left precuneus activation when familiar metaphoric sentences were contrasted with literal sentences (Schmidt et al., 2010). Data from another fMRI study indicated that the right precuneus plays an important role in processing novel metaphors but not in processing familiar metaphors (Mashal et al., 2005), suggesting that it involves retrieval of information from long-term episodic memory or the use of mental imagery. This interpretation is in line with Lakoff and Johnson’s (1980) idea that metaphoric language comprehension may depend on conceptualizations of personal experiences that are stored in episodic memory. Studies with patients with schizophrenia reported other findings. For instance, Kircher et al. (2007) found that literal sentences elicited greater activation in the left and right precuneus relative to metaphoric sentences. Our previous work documented increased activation in the left precuneus during processing of both literal expressions and novel metaphors in people with schizophrenia relative to healthy control participants (Mashal et al., 2013). This means that the precuneus appears to be involved not only in metaphor processing but also in processing of literal language. People with schizophrenia appear to recruit the precuneus but the exact role of the precuneus in language processing in this population remains unclear.

The aim of the present study is to define the role of the precuneus/SPL in processing of metaphors in schizophrenia by applying region-of-interest (ROI) analysis to bilateral precuneus/SPL and language regions. Furthermore, the focus is on precuneus/SPL activation and connectivity. We used a functional connectivity method that measures the interaction of one brain region with another. We thus measured the functional connectivity of the precuneus/SPL with language brain regions (IFG, PSTS) with which it is connected (Cavanna and Trimble, 2006). We also explored whether comprehension of conventional and novel metaphors is associated with signal change in the precuneus/SPL. We hypothesized that the precuneus/SPL would be more strongly activated when participants with schizophrenia processed literal language and novel metaphors relative to healthy participants. Furthermore, we expected to find a correlation between precuneus/SPL response and activation in language brain regions in schizophrenia that would attest for compensation of deficient metaphoric language processing. We also expected to find a positive correlation between signal change in the precuneus/SPL and comprehension of both conventional and novel metaphors.

## Method

### Participants

Twelve outpatients with schizophrenia (mean age = 28.08, *SD* = 4.34) and 12 healthy volunteers (mean age = 27.08, *SD* = 4.10) took part in this research. All participants were native Hebrew speakers and right handed according to self-report. The patient group included five women and had a mean of 12.3 years of formal education (*SD* = 1.3), and the control group included seven women and had a mean of 13.1 years of formal education (*SD* = 1.0). There were no statistically significant group differences in age (*t*_(22)_ = 0.58, *ns*), gender (χ^2^ = 0.67, df = 1, *ns*), or education (*t*_(22)_ = 1.01, *ns*). Patients were recruited through the Tel Aviv Brull Community Mental Health Center, Israel. Two certified psychiatrists verified diagnoses according to the guidelines of the Structured Clinical Interview of the DSM-IV (SCID), Axis I, Patient Edition (First et al., 1994).

Prior to the imaging session, patients were clinically assessed with the Positive and Negative Syndrome Scale (PANSS; Kay et al., 1987) by a clinically trained person. The total mean PANSS score was 58.83 (*SD* = 12.55), with a score of 11.75 (*SD* = 4.29) for positive symptoms, 17.00 (*SD* = 6.95) for negative symptoms, and 30.08 (*SD* = 5.53) for general symptoms. All participants were on stable doses of atypical antipsychotic medication (mean chlorpromazine equivalents = 440 mg/day). Participants received a full explanation of the nature of the study as well as its potential risks and benefits and then provided written informed consent. The study was approved by the Institutional Review Board of Tel Aviv Sourasky Medical Center.

In the present study we reanalyzed the data collected by Mashal et al. (2013) in which 14 people with schizophrenia and 14 healthy participants were scanned. Two participants in each group showed no significant activation in the precuneus/SPL and were thus excluded from the present study.

### Behavioral testing

Participants completed a multiple-choice metaphor comprehension questionnaire. The questionnaire included 30 word pairs: 10 conventional metaphors, 10 novel metaphors, and 10 meaningless expressions (Mashal et al., 2013). For each word pair, four interpretations were provided: a correct interpretation, a literal distracter, an unrelated interpretation, and a phrase saying: “this expression is meaningless”. Participants were instructed to select the best response. The questionnaire was administered after the fMRI session.

### fMRI experiment

Data collection was described in Mashal et al. (2013). Here we reanalyzed the data using a ROI analysis and functional connectivity approaches that were based on the extraction of the individual time courses. To provide the reader with all necessary details, we describe all relevant experimental information from our previous paper.

### Stimuli

We selected 96 Hebrew word pairs that formed four types of semantic relations: literal (*birth weight*), conventional metaphors (*sealed lips*), novel metaphors (*pure hand*), or unrelated (*grain computer*). Several pretests were performed prior to the study. The aim of the first pretest was to determine whether each two-word expression was literal, metaphoric, or meaningless. Twenty healthy judges saw a list of expressions and were asked to decide if each expression is literally plausible, metaphorically plausible, or unrelated. For each condition we selected expressions that were rated by at least 75% of the judges as literally or metaphorically plausible, or as meaningless. To distinguish between conventional and novel metaphors, another group of 10 judges saw a list of only the plausible metaphors from the first pretest. They were asked to rate the degree of familiarity of these expressions on a 5-point scale ranging from 1 (highly unfamiliar) to 5 (highly familiar). Expressions with a score higher than three were considered conventional (average rating 4.67), whereas expressions with a score lower than three on the familiarity scale were considered novel metaphors (average rating 1.98). The third pretest assessed subjective rating of word frequency. Thirty-one additional raters were asked to rate all words on a 5-point scale ranging from 1 (infrequent) to 5 (highly frequent). The average rating was 3.45 for literal expressions, 3.79 for conventional metaphors, 3.67 for novel metaphors, and 3.38 for unrelated word pairs.

### Experimental task and procedure

The stimuli were presented in a block design fashion. Each block contained six word pairs in one of the experimental conditions. Each word pair was presented for 3000 ms followed by a 500 ms blank. The blocks were separated by either 6 s or 9 s, in which participants viewed a fixation point on a gray background (baseline). Each experimental condition appeared four times (with a total of 16 blocks) during each scan session. Each block contained one distracter, so that within a block of literal word pairs (or conventional or novel metaphors) there was one expression that was meaningless, and within the block of unrelated word pairs appeared one metaphoric expression. The first 18 s of the scan were excluded to allow for T2* equilibration effects.

Participants were asked to silently read each word pair and decide whether the word pair made sense. Prior to the fMRI scan the task was practiced with stimuli that were not used in the experiment.

### Image acquisition

Imaging measurements were acquired through a 3T GE scanner (GE, Milwaukee, WI, USA). All images were acquired using a standard quadrature head coil. The scanning session included anatomical and functional imaging. A 3D spoiled gradient echo (SPGR) sequence with high resolution (a slice thickness of 1 mm) was acquired for each person, in order to allow volumetric statistical analyses of the functional signal change and to facilitate later coordinate determinations. The functional T2* weighted images were acquired using gradient echo planar imaging pulse sequence (TR/TE/flip angle = 3000/35/90) with FOV of 200 × 200 mm^2^, and acquisition matrix dimensions of 96 × 96. Thirty-nine contiguous axial slices with 3.0 mm thickness and 0 mm gap were prescribed over the entire brain, resulting in a total of 159 volumes (6201 images).

### Imaging data analysis

The fMRI data were processed through BrainVoyager software (Version 4.9; Brain Innovation, Maastricht, The Netherlands). Prior to statistical tests, motion correction, high frequency temporal filtering (0.006 Hz), and drift correction (no head movement > 1.5 mm was observed in any participant) were applied to the raw data. Pre-processed functional images were incorporated into the 3D datasets through tri-linear interpolation. Images were smoothed with a 6-mm fullwidth, half-maximum (FWHM) Gaussian filter. The complete dataset was transformed into Talairach space (Talairach and Tournoux, 1988). To allow for T2* equilibrium effects, the first six images of each functional scan were excluded.

### ROIs analyses

Our ROIs were defined anatomically and functionally. Specific effects were studied in the left and right precuneus extending laterally to the superior parietal lobule (SPL) and in pre-determined regions that are part of the language network: the left and right IFG, the left MFG, and the left and right PSTS. Anatomic definition of ROIs was based on sulci and gyri. The precuneus/SPL (BA 7) is limited anteriorly by the cingulate sulcus, posteriorly by the medial portion of the parieto-occipital fissure, and inferiorly by the subparietal sulcus and the intraparietal sulcus; the pars triangularis (BA 45/46) in the IFG (left and right), and the area near or at the PSTS between the superior temporal gyrus and the MTG BA 22 (left and right). Our ROIs were also functionally selected by calculating three-dimensional statistical parametric maps, separately for each participant, using a general linear model in which all three meaningful experimental conditions (literal expressions, conventional metaphors, novel metaphors) were positive predictors, and resting state was a negative predictor, with an expected lag of 6 s (accounting for the hemodynamic response delay). Thus, for each participant, task related activity within the pre-determined regions was identified by convolving the boxcar function with a hemodynamic function (HRF). Table 1 presents the average Talairach coordinates of each ROI in each group.

**Table 1:** Mean Talairach coordinates of activation clusters in regions of interest (ROIs).

ROI	Left	Right
Precuneus/SP (BA7)	(−29, −56, 43)	(10, −70, 36)
PSTS (BA22)	(−54, −48, 6)	(48, −19, 1)
IFG (BA45/46)	(−48, 16, 18)	(46, 23, 5)*
MFG (BA46)	(−45, 28, 25)**	–

* healthy participants only; ** patients only; SP = superior parietal; IFG = inferior frontal gyrus; PSTS = posterior superior temporal sulcus; MFG = middle frontal gyrus.

Time courses of statistically significant voxels were collected in each of the ROIs for each person. Individual averaged MR signals were calculated from all epochs (blocks) of the same condition per activated ROI. Signals were then transformed into percent signal change (PSC) relative to baseline. For all analyses involving the fMRI signal extracted from the ROIs, cluster size involved at least 50 voxels, and the significance threshold was set at *p* < 0.01, uncorrected. Significance tests were thus performed on the average PSC obtained within the cluster of all ROIs, as determined for each condition. Because we examined seven predefined ROIs, we set a more conservative threshold of *p* = 0.007 (calculated as 0.05/7) to account for multiple comparisons. The statistical analyses were conducted with STATISTICA software (version 5).

### Functional connectivity analysis

Functional connectivity analyses were performed by computing pair-wise correlations between activation in the precuneus/SPL and activation in language regions (PSTS, IFG). For each participant, fMRI time series (one for each ROI) were averaged separately across voxels within these ROIs for each type of semantic relation (literal word pairs, conventional metaphors, and novel metaphors). Pair-wise Pearson correlation coefficients were computed between each pair of regions (left precuneus/SPL-left PSTS, right precuneus/SPL-right PSTS, left precuneus/SPL-left IFG), using the averaged time series across participants (for each group and condition) during task performance (excluding the between-blocks intervals). Next, we standardized these signals by subtracting them from the mean activation and dividing by the SD, highlighting the specific condition fluctuations (see also Ionta et al., 2014). The significance of the correlations was evaluated through a random permutation test (for similar bootstrapping analysis see Arzouan et al., 2007). In this test, Pearson correlation coefficients are calculated from 5,000 random permutations of the averaged time courses, and are then used to construct the distribution and test the significance of the original correlation value. Additional correction was used to compensate for the multiple comparisons (2 groups × 3 semantic relations × 3 pairs of regions), resulting in a conservative threshold of *p* = 0.002 (calculated as 0.05/18).

#### The relation between metaphor comprehension and precuneus/SPL activation

Next, we evaluated the correlation between behavioral scores on the metaphor comprehension questionnaire and precuneus/SPL activation. We thus calculated Pearson correlations between the PSC elicited by each metaphoric condition (conventional and novel metaphors) and the scores obtained in the metaphor questionnaire, separately for each participant. Then, we tested the significance of these correlations with a random permutation test (Arzouan et al., 2007) that generated 5,000 random permutations for each condition. This method relies on minimal assumptions and can be applied when the assumptions of a parametric approach are untenable (Nichols and Holmes, 2002). The 5,000 permutations were used to construct the distribution, and test the significance of the original correlation value with a *p* value of 0.006 corrected for multiple comparisons (0.05/8 comparisons = 2 groups × 2 semantic relations × 2 pairs of regions).

## Results

### Behavioral results

**Figure 1:**
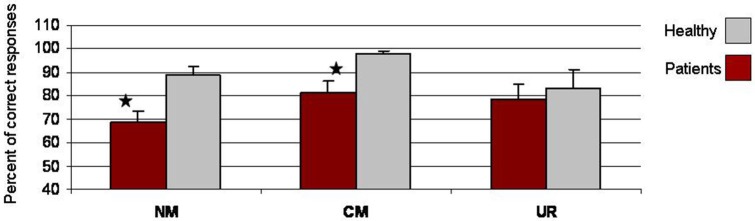
**Mean percent (and standard deviation) of correct responses on metaphor questionnaire, by group**. CM = conventional metaphors; NM = novel metaphors; UR = unrelated word pairs. * denotes *p* < 0.05.

*Metaphoric questionnaire*: People with schizophrenia understood fewer conventional metaphors (mean = 81.25%, *SD* = 18.07) than did healthy individuals (mean = 97.92%, *SD* = 4.8), *t*_(22)_ = 3.08, *p* < 0.01, and fewer novel metaphors (mean = 68.73% correct, *SD* = 17.10) than did healthy individuals (mean = 88.96%, *SD* = 11.82), *t*_(22)_ = 3.42, *p* < 0.01. No significant group difference was found in comprehension of meaningless word pairs (*p* > 0.05). Figure 1 presents questionnaire responses by type of expression and group.

### ROI analysis

Average PSC was analyzed in each of the ROIs by a two-way repeated measures ANOVA in regions showing significant activation by both groups or by a one-way repeated measures ANOVA in regions in which there was significant activation in only one group (see Table 1).

**Figure 2:**
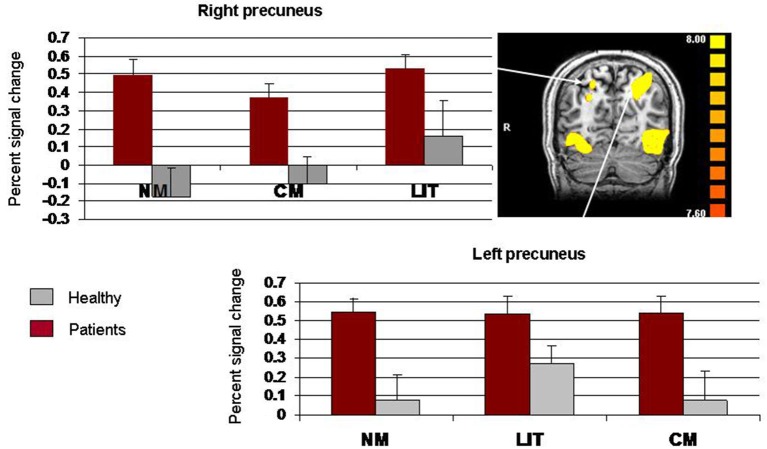
**Whole-brain activation showing signal change for the three conditions (LIT = literal word pairs, CM = conventional metaphors, NM = novel metaphors), vs. baseline using fixed effects analysis (*p* < 0.0001, uncorrected) and percent signal change (SE) in right precuneus/superior parietal lobe**.

A two-way repeated measures ANOVA for signal change within the right precuneus/SPL, with the two groups (schizophrenia, healthy) as a between-subject factor and expression type (literal, conventional, novel) as a within-subject factor, revealed a main effect of group, *F*_(1, 22)_ = 9.29, *p* = 0.006. A Scheffe *post hoc* analysis revealed greater signal change in the schizophrenia group than in the healthy group, *p* < 0.01. The main effect of expression type was also significant, *F*_(2, 44)_ = 8.74, *p* = 0.006. A Scheffe *post hoc* analysis indicated that literal expressions led to greater signal change than did both conventional metaphors, *p* < 0.01, and novel metaphors, *p* < 0.05. However, the group X expression type interaction was not significant, *F*_(2, 44)_ = 3.55, *p* = 0.007 (see Figure 2). A two-way repeated measures ANOVA for signal change within the left precuneus/SPL, with the two groups (schizophrenia, healthy) as a between-subject factor and expression type (literal, conventional, novel) as a within-subject factor, revealed no significant effects (*p*s > 0.007).

**Figure 3:**
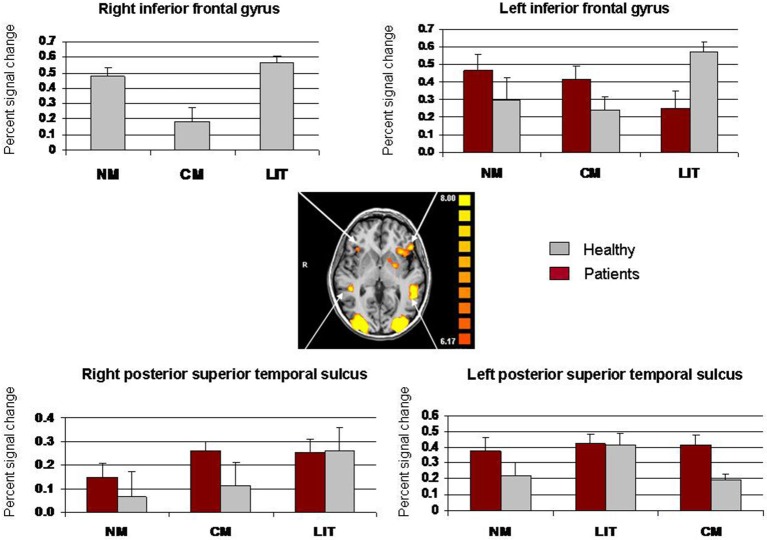
**Whole-brain activation showing signal change for the three conditions (LIT = literal word pairs, CM = conventional metaphors, NM = novel metaphors) vs. baseline using fixed effects analysis (*p* < 0.0001, uncorrected) and percent signal change (SE) in left IFG, right IFG, left PSTS, and right PSTS**.

Percent signal change in the right and left PSTS and the right and left IFG are presented in Figure 3. A two-way repeated measures ANOVA for signal change within the right PSTS with the two groups (schizophrenia, healthy) as a between-subject factor and expression type (literal, conventional, novel metaphors) as a within-subject factor, revealed a main effect of expression type, *F*_(2, 44)_ = 6.27, *p* = 0.004. A Scheffe *post hoc* analysis indicated that literal expressions led to greater signal change than did novel metaphors, *p* < 0.01 (Figure 3). No other effects reached significance (*p* > 0.007). A two-way repeated measures ANOVA for signal change within the left PSTS revealed no significant main effects (*p* > 0.007).

Significant activation in the right IFG was seen in healthy participants alone and therefore a one-way repeated measures ANOVA was performed on signal change in this location, with expression type (literal, conventional, novel) as a within-subject factor. This analysis revealed a significant main effect of expression type, *F*_(2, 22)_ = 10.01, *p* = 0.0008. Signal change for conventional metaphors was significantly weaker than was signal change for novel metaphors, *p* < 0.05, and significantly weaker than was signal change for literal expressions, *p* < 0.01. A two-way repeated measures ANOVA for signal change within the left IFG, with group as a between-subject factor and expression type as a within-subject factor, revealed a significant interaction, *F*_(2, 44)_ = 8.85, *p* = 0.0006. A Scheffe *post hoc* analysis showed that literal expressions led to greater signal change in healthy participants than it did in people with schizophrenia, *p* < 0.05. All other effects were not significant (*p* > 0.007).

**Figure 4:**
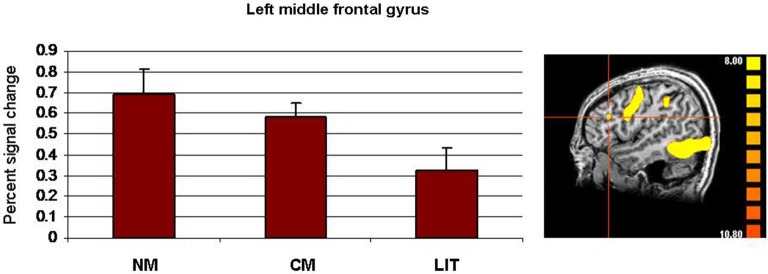
**Whole-brain activation of people with schizophrenia showing signal change for the three conditions (LIT = literal word pairs, CM = conventional metaphors, NM = novel metaphors) vs. baseline using fixed effects analysis (*p* < 0.0001, uncorrected) and percent signal change (SE) in left MFG**.

Finally, because only people with schizophrenia showed significant activation in the left MFG, a one-way ANOVA was performed on signal change in this location, with expression type as a within-subject factor (literal, conventional, novel). No significant main effect of expression type was found (*p* > 0.007) (see Figure 4).

### Functional connectivity analysis

To determine connectivity patterns we calculated pair-wise Pearson correlations between activation in the precuneus/SPL and activation in language regions for each expression type, separately for each group (see Table 2).

**Table 2:** Pair-wise Pearson correlations between activation in precuneus/SPL and activation in pre-determined ROIs, by expression type and group.

		Novel metaphors	Conventional metaphors	Literal expressions
Right precuneus/SPL-Right PSTS	Schizophrenia	0.49*	0.50*	0.37*
	Healthy	0.27	0.17	0.24
Left precuneus/SPL-Left PSTS	Schizophrenia	0.65*	0.62	0.59*
	Healthy	0.42	0.29	0.37
Left precuneus/SPL-Left IFG	Schizophrenia	0.65*	0.63	0.51
	Healthy	0.61	0.52	0.51

* = statistically significant association at the α = 0.0001 level using permutation test.

*People with schizophrenia*: A permutation test analysis showed a significant correlation between activation in the right precuneus/SPL and activation in the right PSTS for literal word pairs, *p* < 0.001, conventional metaphors, *p* < 0.0001, and novel metaphors,* p* < 0.0001. The correlations between activation in the left precuneus/SPL and activation in the left PSTS were significant for both literal word pairs, *p* < 0.0001, and novel metaphors, *p* < 0.00001. There was also a significant correlation between activation in the left precuneus/SPL and activation in the left IFG, but only for novel metaphors, *p* < 0.0001. These results point to left precuneus/SPL involvement in processing of both literal expressions and novel metaphoric expressions and literal word pairs and between activation in the right precuneus/SPL and activation in the right PSTS while processing all semantic relations.

*Healthy group*: No significant correlation between precuneus/SPL activation and activation in the other ROIs was observed within the control group.

### Correlations between performance on the metaphor questionnaire and activation in the precuneus/SPL

To further examine whether metaphor comprehension is related to precuneus/SPL activation, we calculated the correlation between scores on the metaphor questionnaire and the BOLD signal recorded within the left and the right precuneus/SPL.

*People with schizophrenia*: using the permutation test, the only correlation that was found to be significant was the correlation between the comprehension of novel metaphors and BOLD signal in the right precuneus/SPL, *r* = 0.83, *p* < 0.001. Thus, the more correct responses that were given on the questionnaire, the stronger was the BOLD signal within the right precuneus/SPL.

*Healthy participants*: No significant correlations between questionnaire score and precuneus/SPL activation were found in the control group.

## Discussion

The purpose of the present study was to examine the role of the precuneus/SPL in metaphor comprehension in schizophrenia. Three main findings emerged: (1) people with schizophrenia showed greater activation in the right precuneus/SPL relative to healthy participants; (2) within the schizophrenia group BOLD signal in the left precuneus/SPL and in the left PSTS correlated positively during comprehension of both literal word pairs and novel metaphors. There was also a positive correlation between activation in the right precuneus/SPL and in the right PSTS in all semantic relations. In addition, the left precuneus/SPL was co-activated with the left IFG during novel metaphor processing. No equivalent correlations with activation in the precuneus/SPL were found in the healthy group; and (3) within the schizophrenia group comprehension of novel metaphors, as measured by an off-line questionnaire, was correlated with increased activation in the right precuneus/SPL.

The behavioral results showed that people with schizophrenia understood fewer metaphors than did healthy participants. This reduced accuracy is consistent with previous evidence of difficulties in metaphor comprehension in schizophrenia (e.g., Iakimova et al., 2005; Kircher et al., 2007), and is associated with an abnormal pattern of brain activation in schizophrenia (Kircher et al., 2007; Mashal et al., 2013). The present study suggests that the right precuneus/SPL is involved in processing linguistic expressions in schizophrenia, and in particular in understanding novel metaphors. People with schizophrenia may recruit this right posterior parietal region to compensate for their deficient metaphor comprehension. It is also possible that metaphor comprehension is deficient in schizophrenia because this area is recruited. However, the current study cannot determine which explanation is correct. Our results also show that increased novel metaphor comprehension (as assessed by the off line questionnaire) was correlated with increased activation in the right precuneus/SPL, consistent with previous views about the central role of the right hemisphere in metaphor comprehension (Bottini et al., 1994; Giora, 1997, 2003; Mashal et al., 2005, 2007).

The precuneus/SPL has been linked to both linguistic and cognitive processes. According to recent meta-analyses, the precuneus is part of the brain networks associated with semantic processing (Binder et al., 2009; Price, 2010). Our findings point to increased activation in the right precuneus/SPL in schizophrenia as compared to controls. It is possible that this increased activation reflects the process of linking two words into a meaningful expression. However, because processing novel metaphors is demanding, requiring the extraction of relevant features of two disparate domains (Bowdle and Gentner, 2005), greater activation is expected when we compare novel metaphors to literal expressions. Nevertheless, the results of the ROI analysis documented similar signal change across different semantic relations. Hence, it is less likely that precuneus/SPL activation reflects semantic processing in general (Binder et al., 2009). Following Lakoff and Johnson (1980), we assume that people construct mental images in order to use and understand not only figurative language but also literal language. The way in which people construct these mental images differs between the two types of expressions though. While the mental images invoked by figurative language are constrained by conceptual mappings between the base and target domains, the mental images invoked by literal language are based on the understanding of basic level prototypes. Thus, it is possible that people with schizophrenia, unlike healthy participants, either use the right precuneus/SPL to form mental images for both literal and figurative language. Alternatively, it is possible that people with schizophrenia may engage in retrieval of personal experiences from episodic memory (Lakoff and Johnson (1980)).

The low activation observed in the right precuneus/SPL within the healthy group may be related to the observation that the precuneus is part of the DMN (e.g., Fransson, 2005; Cavanna and Trimble, 2006; Fransson and Marrelec, 2008; Margulies et al., 2009; Zhang and Li, 2012). Indeed, there is evidence suggesting that the precuneus is normally less activated during attention-demanding tasks (Cabeza and Nyberg, 2000). It is therefore possible that the pattern of right precuneus/SPL activation in the control group reflects reliance on attentional resources during metaphor processing. It is also possible that the increase in activation in the schizophrenia group is due to abnormalities in the resting state network. Bluhm et al. (2007) reported altered spontaneous fMRI signal fluctuations in the precuneus/posterior cingulated cortex in schizophrenia during resting state. Thus, our results may suggest that whereas healthy participants activate the right precuneus/SPL in accordance with its role as a central hub in the DMN, people with schizophrenia fail to use these attentional resources.

Functional connectivity analyses allowed us to detect associations between neural regions that conventional activation-based analyses cannot address. An important finding of our study is the strong functional connectivity between the left precuneus/SPL and the left PSTS during comprehension of literal word pairs and novel metaphors, as well as the strong connectivity between the right precuneus/SPL and the right PSTS during processing of all semantic relations. These findings suggest that the interactions between the precuneus/SPL and the posterior language area, PSTS, may serve to mediate metaphor comprehension in schizophrenia. The fact that the left precuneus/SPL and the left PSTS, to which the precuneus has anatomical connections (Cavanna and Trimble, 2006), were correlated during literal language comprehension as well indicates that people with schizophrenia may automatically activate mental images in response to both literal and metaphoric expressions (Lakoff and Johnson (1980)). The mental images may then be transformed to auditory representations in the left PSTS to enhance comprehension. In addition, the fact that the left precuneus/SPL was co-activated with the left IFG during novel metaphor comprehension suggests that people with schizophrenia use this brain region in collaboration with the IFG to facilitate novel metaphor comprehension. As argued by Lundstrom et al. (2005), this co-activation may reflect reliance on previous contextual associations which are processed in the precuneus/SPL and their integration in the left IFG. Thus, our results may explain some of the inconsistency in previous fMRI studies in which both left and right precuneus involvement was seen in processing of both literal language and metaphors (e.g., Kircher et al., 2007; Schmidt et al., 2010; Mashal et al., 2013). We suggest that people with schizophrenia, but not healthy participants, use the bilateral precuneus/SPL in collaboration with language areas to facilitate both literal and novel metaphor comprehension.

The current ROI analysis revealed abnormal patterns of signal change in people with schizophrenia. Whereas the right IFG was activated in the healthy group, no such activation was recorded in the schizophrenia group. Thus, consistent with the right hemisphere hypothesis (Mitchell and Crow, 2005), lateralization patterns were different in this group. Interestingly, the ROI analysis found greater activation for processing literal expressions as well as novel metaphors relative to conventional metaphors. This finding indicates that the right lateralized activation observed in healthy individuals was not limited to the interpretation of figurative language but included literal language as well. Unlike the group difference that was documented in the right IFG, both groups activated the left IFG. The ROI analysis demonstrated that the healthy group had greater activation in the left IFG while processing literal word pairs than did the schizophrenia group. Thus, whereas both groups activate the left IFG during metaphor processing to the same extent, the patients show deficient activation of the right IFG.

There are some limitations to our study. First, a larger sample size would have strengthened our conclusions. Given that evidence from different analyses converge in showing involvement of the right precuneus/SPL in novel metaphor processing in schizophrenia, we believe the results will be replicated with a larger group of patients. However, a larger sample size of healthy participants is required to test whether the lack of significant connectivity seen in this group stems from the small sample of healthy participants. Second, we performed the analyses on a subgroup of 24 participants (out of 28 in the original study) who showed significant activation within selected ROIs. It is thus possible that the activation pattern seen here is not universal. We note that the exact role of the precuneus/SPL in language processing is still not entirely clear. However, if the precuneus/SPL activates mental images in response to the current task then we expect to see activation in this area during performance of tasks that explicitly tap into the mental visualization of linguistic expressions. Furthermore, because the expressions used in the current study form a continuum in terms of literality and abstractness (Laor, 1990), people with schizophrenia evoke different mental images on that continuum. Future studies are needed to shed more light on the type of mental images processed by the precuneus. Finally, we did not control for medication effects. Although there is evidence that atypical antipsychotic medication enhances cognitive performance (e.g., Sumiyoshi et al., 2001) and specially attention and verbal fluency (Meltzer and McGurk, 1999), the effects of medication on metaphor processing remain unclear.

In summary, our results shed light on precuneus/SPL involvement in metaphor comprehension in people with schizophrenia. The inefficient processing of metaphors in schizophrenia is related to increased activation in the right precuneus/SPL. It appears that people with schizophrenia recruit the right precuneus/SPL to facilitate novel metaphor comprehension, probably because they rely more on mental imagery and episodic retrieval. Furthermore, people with schizophrenia seem to recruit the bilateral precuneus/SPL while processing novel metaphors, as observed by the co-activation of these regions and both language areas. In contrast, healthy participants seem to rely on the bilateral IFG to process literal expressions and the right IFG to facilitate novel metaphor comprehension. Our results also indicate that the precuneus/SPL contributes to comprehension of literal expressions in schizophrenia, as manifested by tight coupling between the precuneus/SPL and the PSTS during literal language processing.

## Conflict of interest statement

The authors declare that the research was conducted in the absence of any commercial or financial relationships that could be construed as a potential conflict of interest.
